# Incremental Market Behavior Classification in Presence of Recurring Concepts

**DOI:** 10.3390/e21010025

**Published:** 2019-01-01

**Authors:** Andrés L. Suárez-Cetrulo, Alejandro Cervantes, David Quintana

**Affiliations:** 1 Department of Computer Science, Universidad Carlos III de Madrid, Leganés, 28911 Madrid, Spain; 2 Centre for Applied Data Analytics Research, University College Dublin, D04 V2N9 Dublin, Ireland

**Keywords:** ensemble methods, adaptive classifiers, recurrent concepts, concept drift, stock price direction prediction

## Abstract

In recent years, the problem of concept drift has gained importance in the financial domain. The succession of manias, panics and crashes have stressed the non-stationary nature and the likelihood of drastic structural or concept changes in the markets. Traditional systems are unable or slow to adapt to these changes. Ensemble-based systems are widely known for their good results predicting both cyclic and non-stationary data such as stock prices. In this work, we propose RCARF (Recurring Concepts Adaptive Random Forests), an ensemble tree-based online classifier that handles recurring concepts explicitly. The algorithm extends the capabilities of a version of Random Forest for evolving data streams, adding on top a mechanism to store and handle a shared collection of inactive trees, called concept history, which holds memories of the way market operators reacted in similar circumstances. This works in conjunction with a decision strategy that reacts to drift by replacing active trees with the best available alternative: either a previously stored tree from the concept history or a newly trained background tree. Both mechanisms are designed to provide fast reaction times and are thus applicable to high-frequency data. The experimental validation of the algorithm is based on the prediction of price movement directions one second ahead in the SPDR (Standard & Poor’s Depositary Receipts) S&P 500 Exchange-Traded Fund. RCARF is benchmarked against other popular methods from the incremental online machine learning literature and is able to achieve competitive results.

## 1. Introduction

Financial market forecasting is a field characterized by data intensity, noise, non-stationary, unstructured nature, a high degree of uncertainty, and hidden relationships [1], being the financial markets complex, evolutionary, and non-linear dynamical systems [2]. Many approaches try to predict market data using traditional statistical methods. Albeit, these tend to assume that the underlying data have been created by a linear process, trying to make predictions for future values accordingly [3]. However, there is a relatively new line of work based on machine learning, whose success has surprised experts given the theory and evidence from the financial economics literature [4,5,6]. Many of these algorithms are able to capture nonlinear relationships in the input data with no prior knowledge [7]. For instance, Random Forest [8] has been one of the techniques obtaining better results predicting stock price movements [9,10,11,12].

In recent years, the notion of concept drift [13] has gained attention in this domain [14]. The Asian financial crisis in 1997 and, more recently, the great crisis in 2007–2008 have stressed the non-stationary nature and the likelihood of drastic structural or concept changes in financial markets [14,15,16,17,18,19].

Incremental machine learning techniques deal actively or passively [20] with the non-stationary nature of the data and its concept changes [13]. However, the problem of recurring concepts [21,22,23], where previous model behaviors may become relevant again in the future, is still a subject of study. As part of the so-called stability–plasticity dilemma, most of the incremental approaches need to re-learn previous knowledge once forgotten, wasting time and resources, and losing accuracy while the model is out-of-date. Although some authors have started to consider recurring concepts [24,25,26,27,28], the number of contributions focused on the financial forecasting domain is still very limited [29,30]. This might be partially explained by the fact that, in this context, the presence of noise and the uncertainties related to the number of market states, their nature, and the transition dynamics have a severe impact on the feasibility of establishing a ground truth.

Our contribution is an algorithm that deals with gradual and abrupt changes in the market structure through the use of an adaptive ensemble model, able to remember recurring market behaviors to predict ups and downs. The algorithm proposed improves a previous algorithm, namely Adaptive Random Forest (ARF) [31], by being able to react more accurately in the case of abrupt changes in the market structure. This is accomplished through the use of a concept history [21,22,32,33,34], which stores previously learned concept representations. When a structural change is detected, it replaces drifting classifiers with either a new concept model or with a concept extracted from the history, using dynamic time-windows to make the decision. As this concept representation is already trained, our algorithm is able to react faster than its predecessor, which is unable to profit from previous models.

The remainder of the paper is organized as follows. In Section 2, we review related work and approaches. In Section 3, we propose the algorithm RCARF. In Section 4, we describe the experimental design, present our empirical results and discuss their implications. Finally, in Section 5 we conclude with a summary of our findings and future lines of research.

## 2. Related Work

The number of approaches proposed for financial applications is vast. In terms of market price forecasting and trend prediction, these can be approached by looking at fundamental and technical indicators. Even though there is controversy regarding the potential of the latter to produce profitable trading strategies [5,35], the fact is that they are widely used in short-term trading [36]. Kara et al. [37] proposed a set of 10 technical indicators identified by domain experts and previous studies [38,39,40,41,42,43,44]. This approach has been used in more recent works (e.g., [4]). Some of them, such as the work of Patel [12], discretize features based on a human approach to investing, deriving the technical indicators using assumptions from the stock market.

Stock markets are non-stationary by nature. Depending on the period, they can show clear trends, cycles, periods where the random component is more prevalent, etc. Furthermore, stock prices are affected by external factors such as the general economic environment and political scenarios that may result in cycles [12]. Under these circumstances, incremental and online machine learning techniques [28,45] that adapt to structural changes, usually referred to as concept drift [13], are gaining traction in the financial domain [14].

In parallel, ensemble techniques are known for their good performance at predicting both cyclic and non-stationary data such as stock prices [9,12,46]. Ensembles are able to cover many different situations by using sets of learners. If a specific type of pattern reappears after a certain time, some of the trained models should be able to deal with it. These techniques, which are commonly used for trend prediction in financial data, are also one of the current trends of research in incremental learning. Lately, several incremental ensembles have been proposed [47] to deal not only with stationary data and recurring drifts but, also with non-stationary data in evolving data streams [20,22,34,48,49,50,51].

There are different types of concept drift detection mechanisms for handling gradual or abrupt changes, blips or recurring drifts [24,26,27,28,52] that can be used to deal with changes in the market behavioral structure [53]. As opposed to stationary data distributions, where the error rate of the learning algorithm will decrease when the number of examples increases, the presence of changes affects the learning model continuously [54]. This creates the need to retrain the models over time when they are no longer relevant for the current state of the market [15].

In the case of repeated cycles, handling of recurring concepts can help reduce the cost of retraining a model if a similar one has already been generated in the past. Fast recognition of a reappearing model may also improve the overall model accuracy as the trained model will provide good predictions immediately.

Gomes et al. [31] proposed an adaptive version of Random Forest that creates new trees when the accuracy of a participant in the ensemble decreases down to a certain threshold. These trees, considered background learners, are trained only with new incoming data and replace the model that raised a warning when this is flagged as drifting. Their Adaptive Random Forest algorithm (ARF) provides a mechanism to update decision trees in the ensemble and keep historical knowledge only when this is still relevant. However, once a tree is discarded, it is completely removed from memory. In presence of recurring concepts, ARF needs to train the trees from scratch.

Gonçalves et al. [23] proposed a recurring concept drift framework (RCD) that raises warnings when the error rate of a given classifier increases. Their approach creates a collection of classifiers and chooses one based on the data distribution. This data distribution is stored in a buffer of a limited length for each of the classifiers. When there is a warning, the newest data distribution is compared to the data distributions of other stored classifiers, to verify whether the new context has already occurred in the past.

Elwell et al. [20] dealt with recurrent concepts in a similar way. Their approach, Learn++.NSE, keeps one concept per batch, not limiting the number of classifiers. The idea, along the lines of Hosseini et al. [48], is to keep all the accumulated knowledge in a pool of classifiers to be used eventually, if needed. However, this approach suffers from scalability bottlenecks in continuous data streams as it does not prune the list of active classifiers. Other approaches have proposed explicit handling of recurring concepts by checking for similarity [21,22,32,33,34]. These store old models in a concept history for comparison when the current model is flagged as changing.

An alternative approach is the use of Evolving Intelligent Systems (EIS) [55]. These have achieved great results classifying non-stationary time series [19,29,30]. The latest EIS works apply meta-cognitive scaffolding theory for tuning the learned model incrementally in what-to-learn, when-to-learn, and how-to-learn [56]. These have also introduced the ability to deal with recurrent concepts explicitly, beating other methods at predicting the S&P500 [29,30]. In this space, Pratama et al. recently proposed pENsemble [57], an evolving ensemble algorithm inspired by Dynamic Weighted Majority (DWM) [50]. pENsemble counts with explicit drift detection, and it is able to deal with non-stationary environments and handle recurring drifts because of its base classifiers. These have a method that functions as a rule recall scenario, triggering previously pruned rules portraying old concepts to be valid again. However, pENsemble differs from our approach and the rest of the architectures of this work in the fact that it is built upon an evolving classifier. There is still an important gap between EIS and the rest of the literature for data stream classification. Features such as meta-cognition and explicit handling of recurrent concepts are still in an early level of adoption outside EIS. Furthermore, extensive application of EIS to challenging domains as stock market prediction is only starting.

Our proposal, which is described in detail in the next section, applies explicit recurring drift handling for price direction prediction to intra-day market data. The foundations of the algorithm start with the core ideas of ARF [31] as an evolving and incremental ensemble technique. The proposed approach extends these with the capability to store old models in a concept history. These models are subsequently retrieved when they are deemed suitable to improve the predictions of the current ensemble. The approach leverages certain ideas from some of the papers cited above, also including adaptive windowing to compare old and background learners based on buffers of different sizes, depending on the speed of the changes.

## 3. Adaptive Ensemble of Classifiers for Evolving and Recurring Concepts

**Figure 1:**
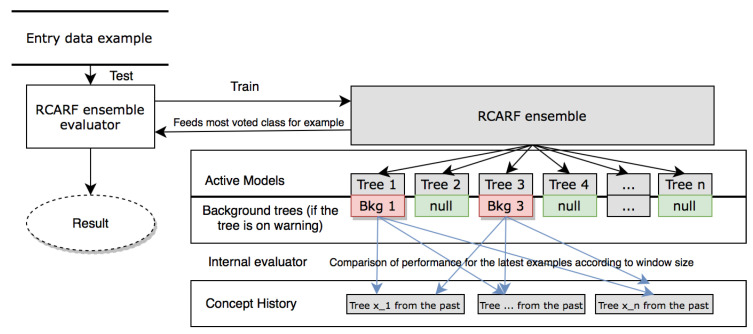
RCARF structure.

The idea behind our proposal, Recurring Concepts Adaptive Random Forest (RCARF), is the development of an algorithm able to adapt itself to gradual, abrupt and also recurring drifts in the volatile data streams of the stock market. The main contribution of the approach is the explicit handling of recurring drifts in an incremental ensemble. This process is managed by two key components: the concept history, and the associated Internal Evaluator. Both are represented in Figure 1, which illustrates the overall structure of the algorithm.

In Algorithm 1, we show the overall pseudocode for the RCARF algorithm. RCARF inherits several features of the Adaptive Random Forest (ARF) algorithm proposed by Gomes et al. [31].

As mentioned above, RCARF is a Random Forest classifier [8]. These algorithms use a collection (ensemble) of “base” classifiers. Traditionally, the forest is a homogeneous set of tree-based classifiers. The full forest classifier performs a prediction for every example in a data stream. Each example or batch of examples is pushed to all the base classifiers, each of which then casts its vote for the most likely class for the example. Each vote is multiplied by the base classifier’s weight, a value that is adapted later depending on whether the related base classifier prediction matches the “real” class of the example. The random component arises from the fact that each of the base classifiers in the ensemble takes into account only a random set of the examples’ features. Even though each base classifier is deciding its individual vote based on partial information, the voting mechanism usually provides very accurate predictions, in many circumstances due to the reinforcing process of the voting mechanism.

This general approach requires some adaptations to handle structural changes on the fly. RCARF implements a basic drift handling strategy along these lines inherited from ARF. To be ready to react properly to structural breaks, these algorithms have a mechanism to detect potential drifts in advance and ensure a smooth transition to new trees. A signal (warning) is raised by a very sensitive drift detector. This triggers the creation of a background tree and starts its training. In the case drift is confirmed (drift signal) at a later stage, the background tree replaces the associated one. Otherwise, it is discarded.

Unlike its predecessor, RCARF is also able to spot the recurrence of previously trained trees and retrieve them from a shared collection of inactive classifiers called concept history. Specific mechanisms in the decision process, such as the internal evaluator, are designed to make the best decision under drift conditions by using only the most adequate sample of recent data.

**Algorithm 1** RCARF algorithm. Adapted from ARF in [31]. Symbols: *m*, maximum features evaluated per split; *n*: total number of trees (n=|T|); $\delta_{w}$, warning threshold; $\delta_{d}$, drift threshold; C(·), change detection method; *S*, data stream; *B*, set of background trees; W(t), tree *t* weight; P(·), learning performance estimation function; CH, concept history; TC, temporal concept saved at the start of warning window. 1:**function** RecurringConceptsAdaptiveRandomForests(m, n, $\delta_{w}$, $\delta_{d}$) 2:  3:    T←CreateTrees(n) 4:  5:    W←InitWeights(n) 6:  7:    B,CH←∅ 8:  9:    **while** HasNext(S) **do**10: 11:        (x,y)←next(S)12: 13:        **for all** t∈T **do**14: 15:           $\hat{y}\leftarrow predict\left(t,x\right)$16: 17:           $W\left(t\right)\leftarrow P(W\left(t\right),\hat{y},y)$18: 19:           RFTreeTrain(m,t,x,y)                           ▹ Train *t* on the current instance(x,y)20: 21:           **if** $C(\delta_{w},t,x,y)$ **then**                                                                  ▹ Warning detected?22: 23:               lastError←evaluate(t)                                             ▹ Save overall error of t24: 25:               TC←copy(t)                 ▹ Copy current tree at the start of warning window26: 27:               b←CreateTree()                                                           ▹ Init background tree28: 29:               B(t)←b30: 31:           **end if**32: 33:           **if** $C(\delta_{d},t,x,y)$ **then**                                                                      ▹ Drift detected?34: 35:               t←bestTransition(t,B(t),CH)36: 37:               addToConceptHistory(TC)                           ▹ Push current concept to CH38: 39:           **end if**40: 41:        **end for**42: 43:        **for all** b∈B **do**                                                            ▹ Train each background tree44: 45:           RFTreeTrain(m,b,x,y)46: 47:        **end for**48: 49:    **end while**50: 51:**end function**

In both adaptive versions of Random Forest, base classifiers are always the Hoeffding Trees used in ARF. That means that, hereafter, we use the term “tree” to refer to each one of these base classifiers. However, it is worth noting that the mechanism we propose does not depend on the type of base classifier, which may be replaced transparently.

For the description of the algorithm, it is important to take into account that every tree generated will be in one of three different states:

- Active trees: Trees currently running in the ensemble for test and train. In the case of drift, an active tree is moved to the concept history.
- Background trees (one per active tree): A new background tree is created when an active tree signals a warning. This starts growing in parallel until a drift is signaled by its active tree (the moment when this gets replaced by either the background tree or a tree from the concept history). We refer to the training time of a background tree as the warning window of its active tree. As in ARF, each background tree inherits warning and drift related parameters values and the maximum subspace size per split from its active tree.
- Concept history trees: These were active trees in the past, but eventually they were replaced because of their low performance in a certain period of time. Throughout this work, when these trees are re-activated, they are called recurring trees.

Code kept from ARF includes the function responsible for inducing each base tree (Algorithm 2) and the warning and drift detection and handling (Lines 1–21, 27–29 and 43–47 in Algorithm 1). The method retains the mechanisms related to the ensemble itself (bagging, weighting and voting). However, in RCARF, we introduce the steps required to manage the concept history and how to perform an informed decision as to how to replace active trees in case of drift (Lines 23–25, and 35–37). These aspects of RCARF are detailed in the sections that follow.

**Algorithm 2** Random Forest Tree Train (RFTreeTrain). Symbols: λ, fixed parameter to Poisson distribution; GP, grace period before recalculating heuristics for split test; m: maximum features evaluated per split; t, decision tree selected; (x, y), current training instance. Adapted from [31]. 1:**function** RFTreeTrain(m, t, x, y) 2:  3:    k←Poisson(λ=6) 4:  5:    **if** k>0 **then** 6:  7:        l←FindLeaf(t,x) 8:  9:        UpdateLeafCounts(l,x,k)10: 11:        **if** examplesSeen(l)≥GP **then**12: 13:           AttempSplit(l)14: 15:           **if** DidSplit(l) **then**16: 17:               CreateChildren(l,m)18: 19:           **end if**20: 21:        **end if**22: 23:    **end if**24: 25:**end function**26: 

### 3.1. Concept History

As stated previously, one of the core elements of RCARF is the addition of a concept history to the ARF schema. The concept history (CH) is a collection of trees shared by all trees in the ensemble. This collection is created during the execution of the algorithm, and is stored for future use when an episode of concept drift impacts the performance of active trees. If an active tree is inserted in the concept history, it becomes available for the whole ensemble. If a tree from the concept history is “promoted” to be an active tree, it is immediately removed from the concept history.

RCARF relies on the assumption that, particularly in the case of abrupt drift, the background tree learned from scratch from the beginning of the warning window may be at a disadvantage compared to an old tree adapted to obtain good results but subsequently discarded. This situation, which would be affected by the speed of the concept drift, is especially likely if we can expect episodes of recurring drift in the data. In that case, the concept history already contains trained trees well-adapted to the recurring concept. Thus, instead of discarding useful trees, the objective would be storing them and then recovering them whenever they become relevant again.

Figure 1 illustrates the structure of RCARF. First, incoming data examples are tested using the ensemble evaluator. Only then, the example is used also for training the active tree.

As stated in the algorithm in Algorithm 1 and by Gomes et al. in [31], when the error statistics increase over time up to a certain threshold, a warning is activated and a background tree is created to replace the active model in the case of drift. After performing these steps, a change detector decides if the algorithm must be prepared for the occurrence of concept drift (warning detection, Line 21) or if a drift has really happened (drift detection, Line 33).

In both ARF and RCARF, the “warning window” is defined as the period of time that starts when an active tree raises a warning and finishes when the same tree detects a drift. Each warning window is specific to an active tree, and resets in the case of false alarm; that is, if a new warning is raised by the same tree before the drift is confirmed. In ARF, if a drift is detected (Line 33), the warning window is finished and the background tree replaces the active tree. In RCARF, during the warning window, there is also an online evaluation on the background tree (the one linked to the active tree that has raised the warning) and all trees in the concept history to compare their performance. This is the task of an “internal evaluator”, described below. Only when a drift is detected, the tree with the lowest error according to the internal evaluator is promoted to active (Line 35). The previously stored copy of the active tree is then moved to the concept history (Line 37).

### 3.2. Internal Evaluator

RCARF has two types of evaluators: the ensemble one and the internal one.

- Ensemble evaluator (global, Line 15): It is in charge of the predicted accuracy and results of the algorithm.
- Internal evaluator (tree-specific, Algorithm 3): This is one of the main components of our proposal. During the warning window, we must collect information to be able to take the best decision of which is the best classifier in case drift happens. Given that we are estimating performance when drift is happening, only the latest examples are relevant. This evaluator is updated every time that a new example is tested. As we can see in Algorithm 4, the internal sliding window changes size dynamically during the warning window only for the background trees. The window size is fixed for trees in the concept history, and it is provided as an input parameter.

**Algorithm 3** Internal evaluator. It computes the best transition in the case of drift. Symbols: *t*, active tree; *b*, background tree; CH, concept history; *c*, tree from CH; WS(CH), fixed window size in CH; WS(b), current window size in *b*, W(c), error statistics in *c* for the latest examples in WS(CH); W(b), error statistics in *b* according to WS(b). 1:**function** BestTransition(*t*, *b*, CH) 2:  3:    **for all** c∈CH **do**                                           ▹ Rank of errors of each tree in CH 4:  5:        addToRank(c,countErrors(W(c))/WS(CH)) 6:  7:    **end for** 8:  9:    **if** minError(rank)≤(countErrors(W(b))/WS(b)) **then**10: 11:        R←extractClassifier(CH,minErrorKey(rank))           ▹ Get and remove tree from the concept history12: 13:    **else**14: 15:        R←b16: 17:    **end if**18: 19:    **return** R20: 21:**end function**22: 

**Algorithm 4** Internal evaluator with dynamic windows for background trees. Symbols: WS, evaluator window size; *W*, evaluator window; SI, size increments; MS, minimum size of window. 1:**function** AddEvaluationResults(value = correctlyClassifies ? 0 : 1) 2:  3:    removeFirstElement(W) 4:  5:    add(W,value)                                 ▹ Add result [1 (error) or 0 (success)] to window 6:  7:    updateWindowSize() 8:  9:    **if** (countOfErrors(W)/WS)<getErrorBeforeWarning **then**10: 11:        WS=WS+SI12: 13:    **else if** WS>MS **then**14: 15:        WS=WS−SI16: 17:    **end if**18: 19:**end function**20: 

The adaptation mechanism for the window size in Algorithm 4 is as follows: if the error obtained by a background tree for its internal evaluator window size (WS) in the latest testing examples is lower than the error obtained by the active tree when it raised the warning signal, then WS decreases down to a minimum size. Otherwise, it increases once per iteration (that is, per example evaluated). Increments and decrements of WS are performed according to an input parameter that defines “size increments” (SI).

The logic of the resizing mechanism relies on the interpretation of the error obtained by the background trees. In cases where it is greater than the error obtained by the active tree before warning, we believe that the underlying reason must be either because the background tree has not been trained with enough samples yet, or because the structure of the data stream is continuously changing (in a period of transition). In the second scenario, a smaller sample of the latest examples could be more accurate in estimating which is the best classifier for the latest concept (WS decreases). Otherwise, a larger sample would be desirable, as it would provide a more representative set of data (WS increases).

### 3.3. Training of the Available Trees

The addition of the concept history and the differences in the replacement strategy used in RCARF entail the need to discuss the way data are used to train the trees. As in ARF, both the active and background trees are trained with new examples as soon as they are available (Lines 19 and 45 in Algorithm 1). However, trees in the concept history are adapted to data that correspond to a different concept. Therefore, they are not retrained unless they are promoted to active.

As mentioned above, in the case of drift, the active tree is replaced by either the best tree from the concept history or the background tree (Lines 35–37 in Algorithm 1) following Algorithm 3. In the case that the background tree was selected for promotion, the training examples from the warning window would already have been used for its training. Conversely, if a concept history tree were selected for promotion, these training examples would be discarded.

As stated by Alippi et al. [21], there is always a delay from the start of a concept drift to the start of the warning window. During this lag, it is not possible to warrant the isolation of a given concept. In this paper, for simplicity, we avoid taking into consideration this delay as a part of our analysis. Therefore, for the purpose of this work, we assumed that the start of every warning window that ends with the trigger of the drift (thus, when this is not a false alarm), matches the start of a concept drift. For this reason, even though active trees are being updated during warning windows, we consider that the moment in which they are best adapted to a given concept is just before the warning window. Hence, the tree that is pushed to the concept history is a snapshot of the active tree at the start of the warning window (see Lines 25 and 37 in Algorithm 1).

## 4. Experimentation: Predicting the S&P500 Price Trend Direction

### 4.1. Data

Data for this work were produced in the following way. First, we downloaded Exchange-Traded Fund (ETF) SPY prices for the entire first quarter of 2017 at second level from QuantQuote (Data source: https://www.quantquote.com). This ETF, one of most heavily-traded ones, tracks the popular US index S&P 500. Secondly, we selected 10 different technical indicators as feature subsets based on the work by Kara et al. [37]. The default value of 10 s that we set for the number of periods, *n*, was extended in the case of the two moving averages. Once we considered the additional possibilities, 5 and 20 s, we ended up with the 14 features described in Table 1. These were computed with the TA-lib technical analysis library (Technical Analysis library: http://ta-lib.org/) using its default values for all parameters other than the time period.

The label, categorized as “0” or “1”, indicates the direction of the next change in the EFT. If the SPY closing price at time *t* is higher than that at time t−1, direction *t* is “1”. If the SPY closing price at time *t* is lower or equal than that at time t−1, direction *t* is “0”. Furthermore, as part of the labeling process, a lag of 1 s has been applied over the feature set. Thus, if the technical indicators belong to the instant t−1, the label reflects the price change from t−1 to *t*.

Short sellers are usually averse to holding positions over non-market hours and try to close them at the end of the day [58]. The price may jump, or the market can behave very differently in the next morning. Therefore, only prices during market hours are considered in this work. In addition, as the technical indicators selected depend on the 35 previous seconds of data, the first 35 s are discarded for each day after processing the technical indicators. This filtering aims to avoid the influence of the previous day trends, and prices before market hours.

### 4.2. Experimental Setting

We designed the experiments presented in this section with two separate purposes in mind.

First, were compared the utility of the recurring drift detection implemented in RCARF vs. the basic ARF approach. To perform a fair comparison between RCARF and ARF, both algorithms used the same ADaptive WINdowing (ADWIN) [59] change detector for warnings and drifts. Furthermore, both learners used the same adapted version of Hoeffding Trees as base classifier, and the same number of trees in their configuration.

Secondly, we aimed to prove that RCARF is a suitable candidate for this task compared to other state-of-the-art learners for data stream classification. For this comparison, we selected the following learners, all of them from the literature of online classification of non-stationary data streams: DWM [50] using Hoeffding Trees as base classifiers, a RCD learner [23] of Hoeffding Trees and Hoeffding Adaptive Tree (AHOEFT) [60]. All of the experiments were performed using the MOA framework [61], which provides implementations of the aforementioned algorithms, in a Microsoft Azure Virtual Machine “DS3 v2” with the Intel(R) Xeon(R) CPU E5-2673 v4 @ 2.30 GHz processor and 14 GB RAM.

ARF is able to train decision trees in parallel with multithreading to reduce the running time. However, in RCARF, multithreading would impact the results because of the addition of the concept history as a shared storage space used by all trees. Thus, in this work, all experiments were run on a single thread. The impact of multithreading is out of the scope of our proposal.

The dataset was modeled as a finite, ordered stream of data, and evaluation and training were performed simultaneously using Interleaved-Test-Then-Train evaluation [61]. In this method, a prediction is obtained for each example, and success or failure is recorded before the example is used for training and adjusting the model.

We evaluated each algorithm using the accumulated classification error at the end of the period. This error was calculated by dividing the number of misclassified examples by the total number of examples. However, this accumulated error was not adequate to compare how different algorithms behave in particular moments of time. Thus, we also calculated at regular intervals the error of each algorithm calculated over a fixed window of time (500 examples). This sequence could then be compared graphically.

Given the stochastic nature of the algorithms based on Random Forests (RCARF and ARF), in these cases, we performed 20 experiments and averaged the results. The statistical significance of the differences of performance among the algorithmic approaches was formally tested using a protocol that starts verifying the normality of the distribution of prediction errors over the mentioned experiments using the Lilliefors test. In the case that the null hypothesis of normality was rejected, we relied on the Wilcoxon test [62]. Otherwise, we tested for homoscedasticity using Levene’s test and, depending on whether we could reject the null hypothesis, the process ends testing for equality of means using either a Welch test or a *t*-test. The significance levels considered in the tests for normality and homoscedasticity were set at 5%. For the rest, we considered both 5% and 1%.

It is worth emphasizing that the approach that we describe predicts short-term market trends, but it does not generate any trading signals (that would require further processing). All the tested algorithms, including our proposed method, used the values of the raw technical indicators at specific points in time to generate a binary class prediction of the price trend (up or stable/down) for new data patterns. Ensemble based approaches have a number of internal classifiers whose predictions are subsequently combined to provide this prediction for the whole ensemble. We emphasize this idea at the end of Section 5.

### 4.3. Parameter Selection and Sensitivity

The parameterization effort was not subject to systematic optimization and, therefore, the performance of the algorithm might be understated. The algorithms in the experiments held most of their respective default parameters or recommended setups according to their authors. Nonetheless, there are certain points common to most of the algorithms that deserve a mention.

- Base learner: All ensembles in our experiments used Hoeffding Trees as base classifier.
- Batch size: The algorithms ARF, RCARF and AHOEFT processed examples one at a time. For RCD, we processed data in batches of 600 examples, that is, 10-min intervals. In DWM, we used its default setup.
- Change detector: All ensembles in our experiments used ADWIN as explicit change detection mechanism. Apart from being the default change detector in ARF, its performance has already been proven in [31].
- Ensemble size: An ensemble size of 40 classifiers was applied by default to RCARF and ARF as this value performed well according to the study in [31]. We used 40 classifiers in all ensembles used in this work.

As mentioned above, in the experiments for ARF and RCARF, as change detector, we used the ADWIN algorithm proposed in [60]. The detailed procedure is described, for instance, in [63]. ADWIN has a variable sized sliding window of performance values. If a drift is detected, the window size is reduced; otherwise, it increases, becoming larger with longer concepts. When used as a drift detector, ADWIN stores two sub-windows to represent older and recent data. When the difference in the averages between these sub-windows surpasses a given threshold, a drift is detected.

The ADWIN change detector uses a parameter, δ, the value of which is directly related to its sensitivity. A large value sets a smaller threshold in the number of changes in the monitored statistic (error rate in our case) that triggers the detection event. Specific values for parameters of this sort are dependent on the signal-to-noise ratio of the specific data stream and may impact the overall performance of the algorithm.

The RCARF algorithm assumes that a background tree is created and starts learning as soon as a change is reported by the ADWIN detector with $\delta_{w}$ sensitivity. This background tree is only upgraded to “active” status when drift is confirmed by a second change detector triggered with $\delta_{d}$ sensitivity. Thus, value for warning ($\delta_{w}$) has to be greater than the value for drift ($\delta_{d}$). Large values were selected for the RCARF change detector ($\delta_{w}$ and $\delta_{d}$) to ensure that concept history trees are given a chance to replace the active tree often enough to detect abrupt changes. These were set to $\delta_{w}=0.3$ and $\delta_{d}=0.15$.

The starting size of the dynamic windows for the internal evaluator was 10 examples, with increments or decrements of 1 example in the background trees, and a minimum size of 5 examples.

Although this should be confirmed by further analysis, our experiments suggest that ARF is in general more sensitive to the values of δ than RCARF. We believe that this can be explained by the fact that, in the case of early detection of a drift or abrupt changes, when the background tree is not yet ready to replace the active model, RCARF can still transition to a recurring decision tree that outperforms the incompletely trained background tree. Because of the sensitivity of ARF to these parameters, we tested three configurations for ARF (two of them, “moderate” and “fast”, recommended by the authors in [31]) that are summarized in Table 2. Regarding RCD, given that it uses a single ADWIN change detector, we selected the same value that was used for drift detection in RCARF.

### 4.4. Global Performance Comparison

Table 3 summarizes the results of the experimental work providing the main descriptive statistics for the accumulated error (%) in predicting the market trend, for all the algorithms on the whole dataset over 20 runs. As can be seen, RCARF obtains the most competitive results. The reported differences were formally tested using the previously described protocol, and all of them were statistically significant at 1%.

The differences between RCARF and ARF are due to the fact that, in some of the abrupt changes, RCARF is able to replace the active tree with a trained tree from its concept history, the performance of which is better than the performance of the background tree used by ARF under the same circumstances. When these gains are over the whole period (including stable periods without concept drift), the final average difference is small. Because of the low signal to noise ratio in this domain, we believe that these small gains in predictive accuracy may create a competitive advantage for any trading system that might use the prediction of RCARF as part of its decision process.

Two configurations of ARF, ARF${}_{moderate}$ and ARF${}_{fast}$, obtained the second- and third-best results, followed by RCD. AHOEFT obtained the worst result, which was expected, as this algorithm maintains a single tree (not an ensemble of trees). It is well-known that ensemble methods can be used for improving prediction performance [64]. We can also conclude that configurations for ARF suggested by the authors are better than ARF${}_{ultra}$, which used the same parameters as RCARF. This may be explained by the fact that this configuration may be too sensitive to noise. It produces too many switches to background trees that are not yet accurately trained when they must be promoted to be active trees. RCARF, instead, is able to switch to pre-trained trees stored in the concept history, thus avoiding the corresponding decrease in performance.

As can be seen in Table 4, RCARF performed an average of 85 drifts per decision tree, for a total of 3411 drifts on average per experiment. However, the final number of decision trees in the concept history was in average 118 trees. As each recurring drift pushes one tree but also deletes one tree from the concept history, the table shows that there were only 118 background drifts in an average experiment, while there were more than 3000 recurring drifts on every experiment. This, together with the obtained results for RCARF, shows that the recurring drift mechanism was used to resolve most of the drift situations.

Another issue of interest is that the final number of active warnings (at the end of the experiments) was between 9 and 19; that is, with 40 trees, a percentage between 22.5% and 50% of the total were in warning at this point of time. Obviously, this fraction changed continuously during the experiment and was different on every run and for each base classifier. This number depends on the sensitivity parameter in RCARF $\delta_{w}$, and may be taken as a measure of the number of “open” warning windows in a given experiment. A lower value for $\delta_{w}$ may be chosen to reduce the number of warning windows opened simultaneously.

In terms of efficiency, the average full running time of RCARF on the whole dataset over 20 experiments was 35,263 s. This is less than the 10 computing hours for an entire quarter of market data at 1-s level. Hence, although the experiments were not run against the market in real time, RCARF demonstrates the ability to operate in an online setting at 1-s level on the server used.

### 4.5. Evolution of the Ensemble over Time

**Figure 2:**
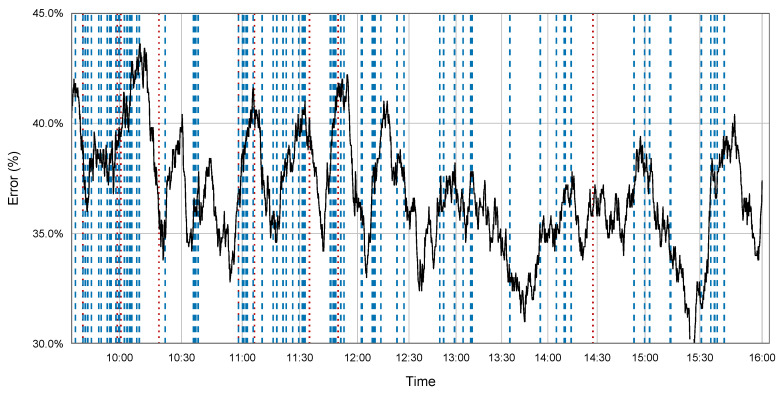
Sample run of RCARF on a single test for the trading first day. Error measured on windows of 500 examples. Red dotted vertical lines mark drifts to background trees, and blued dashed vertical lines mark drifts to recurring trees.

To show the overall behavior of RCARF, we have included Figure 2. It shows the evolution of error in RCARF for a short period of time (the first trading day of the year). Vertical lines are used to signal moments where a drift occurred and an active tree was replaced with one of the trees in the ensemble. Red dotted lines indicate times where a background tree became active, while blue dashed lines indicate times where a concept history tree was re-activated (recurrent drift). As we can observe in Figure 2, drifts are detected throughout the whole period of time.

At the beginning of the experiment, the error was higher because the models had not yet had the opportunity to adjust to the data; therefore, drifts occurred quite often and sometimes with very short intervals among them. Later on, drifts were more sparse. Most of the transitions were to trees that were stored in the concept history (blue dashed drifts in Figure 2), and not very often to background trees (red dotted drifts). That is, concept history trees were used most of the time instead of background trees, which proves that storing information from the past helped the RCARF algorithm in this particular dataset.

**Figure 3:**
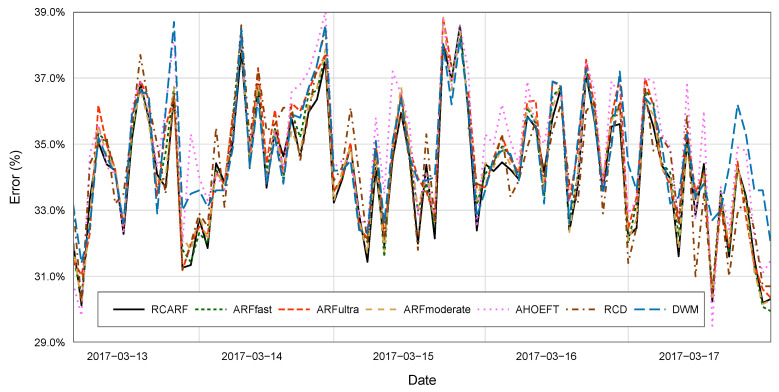
Algorithm comparison. Average error measured on windows of 1000 examples for a example period of time. For RCARF, ARF${}_{ultra}$, ARF${}_{fast}$ and ARF${}_{moderate}$, we show the average result of 20 runs.

Figure 3 compares the results of all of the algorithms over a portion of the training set. Due to the sampling frequency of seconds, we have smoothed the plots averaging error on 1000 examples. The first 1000 examples are excluded from the chart due to this fact. The aim of the figure is to illustrate the performance of the algorithms over a specific period of time. Given the length of the time series used in the experimental analysis and the fact that the algorithms were run a number of times, it is hard to extract clear conclusions out of it. The performance comparison should be made based on the global performance indicators and statistical tests reported Table 3.

Having said that, the figure is consistent with the mentioned results. ARF and RCARF show a similar behavior, and their average error over time tended to be below the one found for the other algorithms. This is interesting because it suggests that these algorithms might indeed be superior under most circumstances, and not under some specific market conditions that might be difficult to capture with the AHOEFT, RCD, and DWM. RCARF and ARF${}_{moderate}$, the closest competitor, often overlapped. However, RCARF was often dominant for short periods of time. This would be consistent with the notion that RCARF should benefit from the use of its concept history to adjust faster to drifts than ARF, which would eventually accumulate enough evidence to converge to a similar model.

## 5. Summary and Conclusions

In this paper, we introduce RCARF, an ensemble tree-based online classifier that handles recurring concepts explicitly. The algorithm extends the capabilities of Adaptive Random Forests (ARF) adding a mechanism to store and handle a shared collection of inactive trees, called concept history. This works in conjunction with a decision strategy that reacts to drift by replacing active trees with the best available alternative: either a previously stored tree from the concept history or a newly trained background tree. Both mechanisms are designed to provide fast reaction times and are thus applicable to high-frequency data.

The experimentation was conducted on data from a full quarter of both price and trade volumes for the SPY Exchange-Traded Fund. This ETF, one of most heavily-traded ones, tracks the S&P 500 index. Both series were downloaded with a resolution of 1-s. We defined a classification problem where the objective was to predict whether the price will rise or decrease in the price change. For this classification task, we used as attributes a list of technical indicators commonly found in the literature. These indicators were labeled with the predicted behavior (class) and the result was fed as a data stream to our test bench of online stream classifiers, including our proposal, RCARF.

The experimental results show that RCARF offers a statistically significant improvement over the comparable methods. Given that the main difference between ARF and RCARF is the fact that the second one uses recurring concepts, the new evidence would support the hypothesis that keeping a memory of market models adds value versus a mere continuous adaptation. The idea that old models might end up eventually being more useful than the ones that are being fitted at the time, mostly due to faster adaptation to the market state, has interesting implications from a financial point of view. The reported results would support the idea of history repeating in terms of the price generation process. The market would not always transition to completely new market states, but also switch back to previous (or similar) ones. Recognition of the previous aspect is an extra insight for financial experts that might be used to obtain excess returns. This, however, is something to be analyzed in the future.

This work was focused on trend prediction with adaptation to concept drift, but we did not intend to derive any trading system. Actually, the implementation of such system might require reframing the classification problem to include a larger number of alternatives that could discriminate not only the direction of price changes, but also their magnitude. The current version of the algorithm predicts to a certain point short-term market trends, whether there is a way to exploit profitably market regularities is yet to be determined. For that reason, while it is clear that our the results are compatible with arguments against the efficient-market hypothesis, we cannot claim that we can beat consistently buy and hold and, therefore, we cannot reject it.

Future extensions of this work might include optimization of the algorithm for ultra-high frequencies and the development of further methods to adapt and resize the internal evaluator, such as the possibility of saving the window size as part of the concept to be inherited in case of recurring drifts and new window resizing politics for the historical models. All this might contribute to the optimization of the process that currently selects between recurrent or new decision trees. Finally, another possibility would be the addition of meta-cognition to evaluate recurring behaviors from the history by looking at previous transitions of the model.

## Figures and Tables

**Table 1:** Selected technical indicators. Formulas as reported in Kara et al. [37] applied to second-level. Exponential and simple moving averages for 5 and 20 s added as extra features.

Name of Indicators	Formulas
Simple n-second moving average (5, 10, 20)	$\frac{C_{t}+C_{t-1}+\ldots +C_{t-n+1}}{n}$
Weighted n-second moving average (5, 10, 20)	$\frac{n\times C_{t}+\left(n-1\right)\times C_{t-1}+\ldots +C_{t-n+1}}{n+(n-1)+\ldots +1}$
Momentum	$C_{t}-C_{t-n}$
Stochastic K%	$\frac{C_{t}-LL_{t-n}}{HH_{t-n}-LL_{t-n}}\times 100$
Stochastic D%	$\frac{\sum_{i=0}^{n-1}K_{t-i}\%}{n}$
RSI (Relative Strength Index)	$100-\frac{100}{1+\left(\sum_{i=0}^{n-1}Up_{t-i}/n\right)/\left(\sum_{i=0}^{n-1}Dw_{t-i}/n\right)}$
MACD (Moving average convergence divergence)	$EMA{\left(12\right)}_{t}-EMA{\left(26\right)}_{t}$
Larry William’s R%	$\frac{H_{n}-C_{t}}{H_{n}-L_{n}}\times 100$
A/D (Accumulation/Distribution) Oscillator	$\frac{H_{t}-C_{t-1}}{H_{t}-L_{t}}$
CCI (Commodity Channel Index)	$\frac{M_{t}-SM_{t}}{0.015D_{t}}$

$C_{t}$ is the closing price; $L_{t}$ the low price; $H_{t}$ the high price at time *t*; EMA exponential moving average, $EMA{\left(k\right)}_{t}$: $EMA{\left(k\right)}_{t-1}+\alpha \times \left(c_{t}-EMA{\left(k\right)}_{t-1}\right)$; α smoothing factor: 2/1+k; *k* is time period of *k* second exponential moving average; $LL_{t}$ and $HH_{t}$ mean lowest low and highest high in the last *t* seconds, respectively; $M_{t}:H_{t}+L_{t}+C_{t}/3$; $SM_{t}:\sum_{i=1}^{n}M_{t-i+1})/n$; $D_{t}:(\sum_{i=1}^{n}|M_{t-i+1}-SM_{t}\left|\right)/n;Up_{t}$ means the upward price change; $Dw_{t}$ means the downward price change at time *t*. *n* is the period used to compute the technical indicator in seconds.

**Table 2:** Sensitivity parameters for the ADWIN change detector in ARF and RCARF.

Configuration	$\delta_{w}$	$\delta_{d}$
ARF${}_{moderate}$	0.0001	0.00001
ARF${}_{fast}$	0.01	0.001
RCARF, ARF${}_{ultra}$	0.3	0.15
RCD	0.15	

**Table 3:** Global comparison. Accumulated error (%) for all algorithms on the whole dataset, sorted from best to worst result. Main descriptive statistics over 20 runs. Differences are significant at 1%.

	Mean	Median	Var.	Max.	Min.
RCARF	34.7533	34.7538	0.0002	34.7791	34.7285
ARF${}_{moderate}$	34.8008	34.8007	0.0002	34.8362	34.7769
ARF${}_{fast}$	34.8309	34.8335	0.0003	34.8591	34.7902
RCD${}_{HOEF}$	35.0469	35.0469	0.0000	35.0469	35.0469
ARF${}_{ultra}$	35.1104	35.1114	0.0002	35.1392	35.0881
DWM	35.2364	35.2364	0.0000	35.2364	35.2364
AHOEFT	35.4661	35.4661	0.0000	35.4661	35.4661

**Table 4:** Internal statistics for RCARF on the whole dataset over 20 runs. # Drifts, number of total drifts during the execution (both recurring and background); Drifts per tree, number of total drifts during the execution (both recurring and background) divided by the ensemble size; # F. Warnings, number of active warnings at the end of the execution; # CH Trees, number of decision trees in the concept history at the end of the execution.

	Mean	Median	Var.	Max.	Min.
# Drifts	3411.1500	3411.5000	3120.2395	3518	3279
Drifts per tree	85.2788	85.2875	1.9501	88	82
# F. Warnings	13.6000	14.0000	9.2000	19	9
# CH Trees	118.2500	119.0000	46.0921	130	106

## References

[1] Huang W., Nakamori Y., Wang S.Y. (2005). Forecasting stock market movement direction with support vector machine. Comput. Oper. Res..

[2] Abu-Mostafa Y.S., Atiya A.F. (1996). Introduction to financial forecasting. Appl. Intell..

[3] Cavalcante R.C., Brasileiro R.C., Souza V.L.F., Nobrega J.P., Oliveira A.L.I. (2016). Computational Intelligence and Financial Markets: A Survey and Future Directions. Expert Syst. Appl..

[4] Hsu M.W., Lessmann S., Sung M.C., Ma T., Johnson J.E. (2016). Bridging the divide in financial market forecasting: Machine learners vs. financial economists. Expert Syst. Appl..

[5] Fama E.F. (1970). Efficient Capital Markets: A Review of Theory and Empirical Work. J. Financ..

[6] Tsaih R., Hsu Y., Lai C.C. (1998). Forecasting S&P 500 stock index futures with a hybrid AI system. Decis. Support Syst..

[7] Atsalakis G.S., Valavanis K.P. (2009). Surveying stock market forecasting techniques—Part II: Soft computing methods. Expert Syst. Appl..

[8] Breiman L. (2001). Random Forests. Mach. Learn..

[9] Ballings M., Van Den Poel D., Hespeels N., Gryp R. (2015). Evaluating multiple classifiers for stock price direction prediction. Expert Syst. Appl..

[10] Booth A., Gerding E., McGroarty F. (2014). Automated trading with performance weighted Random Forests and seasonality. Expert Syst. Appl..

[11] Ładyżyński P., Żbikowski K., Grzegorzewski P., Rutkowski L., Korytkowski M., Scherer R., Tadeusiewicz R., Zadeh L.A., Zurada J.M. (2013). Stock Trading with Random Forests, Trend Detection Tests and Force Index Volume Indicators. Proceedings of the 12th International Conference on Artificial Intelligence and Soft Computing Part II (ICAISC 2013).

[12] Patel J., Shah S., Thakkar P., Kotecha K. (2015). Predicting stock and stock price index movement using Trend Deterministic Data Preparation and machine learning techniques. Expert Syst. Appl..

[13] Tsymbal A. (2004). The Problem of concept drift: Definitions and Related Work.

[14] Das R.T., Ang K.K., Quek C. (2016). IeRSPOP: A novel incremental rough set-based pseudo outer-product with ensemble learning. Appl. Soft Comput. J..

[15] Münnix M.C., Shimada T., Schäfer R., Leyvraz F., Seligman T.H., Guhr T., Stanley H.E. (2012). Identifying States of a Financial Market. Sci. Rep..

[16] Vella V., Ng W.L. (2014). Enhancing risk-adjusted performance of stock market intraday trading with Neuro-Fuzzy systems. Neurocomputing.

[17] Hu Y., Liu K., Zhang X., Xie K., Chen W., Zeng Y., Liu M. (2015). Concept drift mining of portfolio selection factors in stock market. Electron. Commer. Res. Appl..

[18] Silva B., Marques N., Panosso G. Applying neural networks for concept drift detection in financial markets. Proceedings of the CEUR Workshop Proceedings.

[19] Gu X., Angelov P.P., Ali A.M., Gruver W.A., Gaydadjiev G. Online evolving fuzzy rule-based prediction model for high frequency trading financial data stream. Proceedings of the 2016 IEEE Conference on Evolving and Adaptive Intelligent Systems (EAIS).

[20] Elwell R., Polikar R. (2011). Incremental learning of concept drift in nonstationary environments. IEEE Trans. Neural Netw..

[21] Alippi C., Boracchi G., Roveri M. (2013). Just-in-time classifiers for recurrent concepts. IEEE Trans. Neural Netw. Learn. Syst..

[22] Gomes J.B., Gaber M.M., Sousa P.A.C., Menasalvas E. (2014). Mining recurring concepts in a dynamic feature space. IEEE Trans. Neural Netw. Learn. Syst..

[23] Gonçalves P.M., Souto R., De Barros M. (2013). RCD: A recurring concept drift framework. Pattern Recognit. Lett..

[24] Webb G.I., Hyde R., Cao H., Nguyen H.L., Petitjean F. (2016). Characterizing concept drift. Data Min. Knowl. Discov..

[25] Gomes H.M., Barddal J.P., Enembreck F.I., Bifet A. (2017). A Survey on Ensemble Learning for Data Stream Classification. ACM Comput. Surv..

[26] Ramírez-Gallego S., Krawczyk B., García S., Woźniak M., Herrera F. (2017). A survey on data preprocessing for data stream mining: Current status and future directions. Neurocomputing.

[27] Gama J.A. (2013). A Survey on concept drift Adaptation. ACM Comput. Surv.

[28] Ditzler G., Roveri M., Alippi C., Polikar R. (2015). Learning in Nonstationary Environments: A Survey. IEEE Comput. Intell. Mag..

[29] Pratama M., Lughofer E., Er J., Anavatti S., Lim C.P. (2017). Data driven modelling based on Recurrent Interval-Valued Metacognitive Scaffolding Fuzzy Neural Network. Neurocomputing.

[30] Pratama M., Lu J., Lughofer E., Zhang G., Er M.J. (2016). Incremental Learning of concept drift Using Evolving Type-2 Recurrent Fuzzy Neural Network. IEEE Trans. Fuzzy Syst..

[31] Gomes H.M., Bifet A., Read J., Barddal J.P., Enembreck F., Pfharinger B., Holmes G., Abdessalem T. (2017). Adaptive Random Forests for evolving data stream classification. Mach. Learn..

[32] Yang Y., Wu X., Zhu X. (2006). Mining in anticipation for concept change: Proactive-reactive prediction in data streams. Data Min. Knowl. Discov..

[33] Gomes J.B., Menasalvas E., Sousa P.A.C., Szczuka M., Kryszkiewicz M., Ramanna S., Jensen R., Hu Q. (2010). Tracking Recurrent Concepts Using Context. Rough 557 Sets and Current Trends in Computing, Proceedings of the 7th International Conference, RSCTC 2010, Warsaw, Poland, 28–30 June 2010.

[34] Li P., Wu X., Hu X. (2012). Mining Recurring concept drifts with Limited Labeled Streaming Data. ACM Trans. Intell. Syst. Technol..

[35] Lo A.W., Mamaysky H., Wang J. (2000). Foundations of Technical Analysis: Computational Algorithms, Statistical Inference, and Empirical Implementation. J. Financ..

[36] Tay F.E., Cao L. (2001). Application of support vector machines in financial time series forecasting. Omega.

[37] Kara Y., Acar Boyacioglu M., Baykan O.K. (2011). Predicting direction of stock price index movement using artificial neural networks and support vector machines: The sample of the Istanbul Stock Exchange. Expert Syst. Appl..

[38] Diler A. (2003). Predicting direction of ISE national-100 index with back propagation trained neural network. J. Istanb. Stock Exch..

[39] Armano G., Marchesi M., Murru A. (2005). A hybrid genetic-neural architecture for stock indexes forecasting. Inf. Sci..

[40] Huang C.L., Tsai C.Y. (2009). A hybrid SOFM-SVR with a filter-based feature selection for stock market forecasting. Expert Syst. Appl..

[41] Kim K.J. (2003). Financial time series forecasting using support vector machines. Neurocomputing.

[42] Kim K.J., Han I. (2000). Genetic algorithms approach to feature discretization in artificial neural networks for the prediction of stock price index. Expert Syst. Appl..

[43] Yao J., Tan C.L., Poh H.L. (1999). Neural Networks for Technical Analysis: A Study on Klci. Int. J. Theor. Appl. Financ..

[44] Kumar M., Thenmozhi M. Forecasting Stock Index Movement: A Comparision of Support Vector Machines and Random Forest. Proceedings of the 9th Capital Markets Conference on Indian Institute of Capital Markets.

[45] Lughofer E., Angelov P. (2011). Handling drifts and shifts in on-line data streams with evolving fuzzy systems. Appl. Soft Comput. J..

[46] Patel J., Shah S., Thakkar P., Kotecha K. (2015). Predicting stock market index using fusion of machine learning techniques. Expert Syst. Appl..

[47] Krawczyk B., Minku L.L., Gama J., Stefanowski J., Woźniak M., Wó Zniak M. (2017). Ensemble learning for data stream analysis: A survey. Inf. Fusion.

[48] Hosseini M.J., Ahmadi Z., Beigy H. (2012). New management operations on classifiers pool to track recurring concepts. Lecture Notes in Computer Science.

[49] Hosseini M.J., Ahmadi Z., Beigy H. Pool and Accuracy Based Stream Classification: A New Ensemble Algorithm on Data Stream Classification Using Recurring Concepts Detection. Proceedings of the 2011 IEEE 11th International Conference on Data Mining Workshops.

[50] Kolter J.Z., Maloof M.A. (2007). Dynamic Weighted Majority: An Ensemble Method for Drifting Concepts. J. Mach. Learn. Res..

[51] Karnick M., Ahiskali M., Muhlbaier M.D., Polikar R. Learning concept drift in nonstationary environments using an ensemble of classifiers based approach. Proceedings of the 2008 IEEE International Joint Conference on Neural Networks (IEEE World Congress on Computational Intelligence).

[52] Barddal J.P., Gomes H.M., Enembreck F., Pfahringer B. (2017). A survey on feature drift adaptation: Definition, benchmark, challenges and future directions. J. Syst. Softw..

[53] Gama J., Medas P., Castillo G., Rodrigues P., Bazzan A.L.C., Labidi S. (2004). Learning with Drift Detection. Advances in Artificial 605 Intelligence, Proceedings of the 17th Brazilian Symposium on Artificial Intelligence (SBIA 2004), Sao Luis, Maranhao, Brazil, 29 September–1 Ocotber 2004.

[54] Bifet A. (2017). Classifier concept drift Detection and the Illusion of Progress.

[55] Baruah R.D., Angelov P. (2011). Evolving fuzzy systems for data streams: A survey. Wiley Interdiscip. Rev. Data Min. Knowl. Discov..

[56] Sateesh Babu G., Suresh S., Huang G.B. (2011). Meta-cognitive Neural Network for classification problems in a sequential learning framework. Neurocomputing.

[57] Pratama M., Pedrycz W., Lughofer E. (2018). Evolving Ensemble Fuzzy Classifier. IEEE Trans. Fuzzy Syst..

[58] Chen H., Singal V. (2003). Role of Speculative Short Sales in Price Formation: The Case of the Weekend Effect. J. Financ..

[59] Bifet A., Gavaldà R. Learning from Time-Changing Data with Adaptive Windowing. Proceedings of the 2007 SIAM International Conference on Data Mining.

[60] Bifet A., Gavaldà R., Adams N.M., Robardet C., Siebes A., Boulicaut J.F. (2009). Adaptive Learning from Evolving Data Streams. Advances in Intelligent Data Analysis VIII, Proceedings of the 8th International Symposium on Intelligent Data Analysis (IDA 2009), Lyon, France, 31 August–2 September 2009.

[61] Bifet A., Holmes G., Kirkby R., Pfahringer B. (2010). MOA: Massive Online Analysis. J. Mach. Learn. Res..

[62] Wilcoxon F. (1945). Individual Comparisons by Ranking Methods. Biom. Bull..

[63] De Barros R.S.M., Hidalgo J.I.G., de Lima Cabral D.R. (2018). Wilcoxon Rank Sum Test Drift Detector. Neurocomputing.

[64] Rokach L. (2010). Ensemble-based Classifiers. Artif. Intell. Rev..

